# Robustness Elasticity in Complex Networks

**DOI:** 10.1371/journal.pone.0039788

**Published:** 2012-07-10

**Authors:** Timothy C. Matisziw, Tony H. Grubesic, Junyu Guo

**Affiliations:** 1 Department of Civil and Environmental Engineering, University of Missouri, Columbia, Missouri, United States of America; 2 Department of Geography, University of Missouri, Columbia, Missouri, United States of America; 3 Informatics Institute, University of Missouri, Columbia, Missouri, United States of America; 4 Geographic Information Systems and Spatial Analysis Laboratory, College of Information Science and Technology, Drexel University, Philadelphia, Pennsylvania, United States of America; Technical University of Madrid, Italy

## Abstract

Network robustness refers to a network’s resilience to stress or damage. Given that most networks are inherently dynamic, with changing topology, loads, and operational states, their robustness is also likely subject to change. However, in most analyses of network structure, it is assumed that interaction among nodes has no effect on robustness. To investigate the hypothesis that network robustness is not sensitive or elastic to the level of interaction (or flow) among network nodes, this paper explores the impacts of network disruption, namely arc deletion, over a temporal sequence of observed nodal interactions for a large Internet backbone system. In particular, a mathematical programming approach is used to identify exact bounds on robustness to arc deletion for each epoch of nodal interaction. Elasticity of the identified bounds relative to the magnitude of arc deletion is assessed. Results indicate that system robustness can be highly elastic to spatial and temporal variations in nodal interactions within complex systems. Further, the presence of this elasticity provides evidence that a failure to account for nodal interaction can confound characterizations of complex networked systems.

## Introduction

The structural and operational characteristics of many types of networks, particularly those representing physical, biological, chemical and social systems are highly dynamic and subject to constant change [1]–[4]. Networks exhibit periods of growth, decline, adjustment and equilibrium, expressed as changes to their structure (i.e. topology) and use (i.e. the magnitude of interaction or flow between pairs of nodes). As a result, the ability to effectively characterize the robustness of networks to the deletion of arcs and/or nodes is a tremendous analytical challenge requiring the consideration of both the structural and operative states of a network over time.

Much of the research on characterizing network dynamics focuses on structural change of systems over time, emphasizing network growth via preferential attachment [5]–[7] or copying [8], together with scaling [6], [9]–[10], design considerations [11], decline [12] and vulnerability to failure [13]–[15]. Likewise, research that assesses network robustness typically addresses the problem from a structural perspective, emphasizing various measures of connectivity and performance [9], [11], [16]–[18]. As a result, these types of approaches primarily *describe* a structural state of the network, assuming that interaction or flow among all pairs of nodes is equivalent in magnitude and value to the system. However, in most networks, the level of interaction between any pair of nodes can vary in response to changes in the demand or need for interaction between the pair. For instance, the number of commuters between two cities can vary based on cost of travel, time of day, day of week, services available, etc [19]. Individuals in a social network such as Facebook do not require (or want) connectivity with all other individuals in the network. Likewise, every species in a food web does not consume equivalently at every trophic level. Unfortunately, relatively little research has been devoted to understanding how variations in interaction among network nodes can affect a network’s robustness [20]–[22]. One reason for the overwhelming focus on network structure is that measuring interaction among network nodes is a substantial practical and analytical challenge in itself. Fortunately, new scientific developments continue to yield nodal interaction data of increasing resolution and quality, increasing prospects for more sophisticated assessments of network structure and operation [23]–[26]. Thus, many research areas are now better positioned to exploit these spatial relationships in their analysis and move beyond the overgeneralizations inherent to structural analysis. To investigate the hypothesis that network robustness is not sensitive or elastic to the level of interaction (or flow) among network nodes, this paper explores the impacts of network disruption, namely arc deletion, over a temporal sequence of observed nodal interactions for a large Internet backbone system. In particular, a mathematical programming approach is used to identify exact bounds on robustness to arc deletion for each epoch of nodal interaction. Elasticity of the identified bounds relative to the magnitude of arc deletion is then assessed.

Evaluations of network robustness are typically premised on the extent to which a network is impacted by a disruptive or disassembly mechanism, such as those triggering the deletion or loss of network arcs and nodes [12], [14]. Networks that experience lower levels of disruption from such events are considered to be more robust. One common way of modeling a disruptive mechanism is through the deletion of network elements in a manner that is representative of the assumed process (e.g. random deletion of nodes) [12]. Once an element (or a set of elements) is deleted, the resulting impact to the network’s performance (i.e. decreases in efficiency, connectivity, capacity, interaction, etc.) can be assessed [9]. A network’s robustness is therefore intimately linked with the mechanism of disruption assumed to impact the network and how the resulting disruption is measured.

Given the diversity of networks that have been studied, many mechanisms of disruption have been modeled, giving rise to a tremendous range of methodological options for characterizing and interpreting robustness [18], [27]. Murray et al. [27] provide a basic typology of these methods using four broad categories: 1) scenario specific, 2) strategy specific, 3) simulation, and 4) mathematical modeling. Each of these categories is structured to reflect the way that deletion scenarios (sets of arcs and nodes deleted) are identified. Scenario specific methods are focused on analyzing the impact of a single or very limited selection of deletion scenarios that are presumed to be targeted by a deletion mechanism. Modeling the network effects of an airport closure (i.e. nodal deletion) due to local or regional weather conditions is considered a scenario-specific approach in this typology. Strategy specific methods identify scenarios where a deletion mechanism is assumed to logically order and sequentially delete network elements in some fashion. For instance, nodes might be ranked in decreasing order of their perceived value (e.g. based on some structural characteristic such as degree) to the deletion mechanism to establish the sequence of deletion [9], [20]. Similar to the scenario specific methods, strategy specific approaches assume that scenarios selected by the disruptive mechanism can be precisely determined using simple rules and ranking metrics. As a result, both scenario specific and strategy specific methods for evaluating network robustness typically consider a relatively limited set of deletion scenarios. However, this can be problematic, because even in small networks, an enormous range of potential deletion scenarios exist whose contribution to network robustness remains unexplored. As a result, evaluation, comparison and benchmarking of robustness is limited to only those deletion scenarios identified, rather than a broader spectrum of potential scenarios [18], [27]–[29]. Simulation methods attempt to provide a more detailed characterization of robustness by identifying a larger sample of potential deletion scenarios and assessing their relative value. That is, simulation attempts to relax the rules guiding deletion and account for the wide range of scenarios available to a deletion mechanism. However, unless all potential scenarios of deletion are completely enumerated, the full extent of network robustness remains unknown. This is of particular concern since the deletion scenarios to which the network is least robust may not be identified [18], [27]–[29]. To address this issue, mathematical modeling techniques have been developed to establish the exact mathematical bounds on robustness (i.e. robustness to the most disruptive mechanism and the least disruptive mechanism) [22], [28]–[35]. These mathematical programming approaches, widely known as “interdiction” models, have been developed to identify optimal ways of deleting or degrading network elements by explicitly modeling the objective(s) of the deletion mechanism without constraining the order in which the elements are selected for deletion. In other words, it is assumed that a mechanism optimizes its capability for deletion by assessing the simultaneous impact of the deletion scenario on a network’s robustness. In this sense, one bound on robustness can be viewed as the level of disruption caused by a deletion mechanism that optimally targets a set of arcs and/or nodes such that network performance is maximally degraded. That is, no other scenario of deletion targeting the same number of arcs and nodes would result in greater disruption to network performance than the bounding scenario. Since these bounds represent a mathematically optimal benchmark on network robustness, the relative impacts of all other scenarios of arc/node deletion (regardless of the underlying mechanism) can be evaluated consistently and without bias with respect to the bounds. Sadly, little attention has been given to mathematical bounds on robustness since they are extremely difficult to provably identify due to the combinatorics and interdependencies inherent to complex networks [22], [35]. Consider, for example, a network with 400 nodes and a mechanism targeting eight nodes for deletion. Combinatorially, the mechanism in this case would have 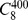 (or 15 quadrillion) feasible scenarios of simultaneous node deletion to choose from. In order to provably identify the scenario bounding robustness of a network this size, the impact of each of these scenarios would need to be evaluated in some fashion. Clearly, while this type of network is not particularly large by today’s standards, the associated computational challenges for evaluating robustness on a system this size are daunting. Regardless, since the mathematical bounds on robustness yield valuable context for robustness measures associated with any other mechanism of deletion, they are ideal for testing the sensitivity of network robustness to changes in nodal interaction as will be examined next. First, a mathematical programming approach for deriving bounds on network robustness to arc deletion given observations of nodal interaction over time is detailed. This modeling framework is then applied to a large Internet backbone system, for which a temporal sequence of nodal interactions was obtained. After robustness bounds are determined, elasticity of the identified bounds relative to the magnitude of arc deletion is assessed.

## Methods

Provided a network *G* with *N* nodes and *A* arcs in epoch *t* (*G_t_(N_t_,A_t_)*) and level of interaction or flow *f_ijt_* between each pair of nodes 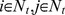, the total nodal interaction supported by the network can be expressed as 
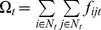
. Let *p* denote the number of arcs to be targeted by the deletion mechanism and let 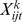 represent the deletion mechanism’s decision to act on each arc 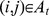 in scenario *k* (where, 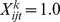 if arc 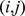 is selected for deletion; 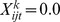 otherwise). Thus, in any feasible scenario of arc deletion *k* in epoch *t*, 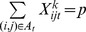. Numerous such scenarios 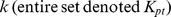 exist given the combinatorial nature of the problem as described earlier. Given any scenario of deletion *k*, the impact on connectivity or the presence of a path between a pair of nodes can then be referenced as the variable 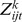 where, 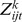 = 0.0 if connectivity between nodes *i* and *j* is present, and 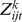 = 1.0 if no path is available between the nodal pair. Thus, interaction between a pair of nodes can only be facilitated whenever *at least* one path between the two nodes exists (i.e. 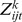 = 0.0). The total nodal interaction inhibited by a scenario of arc deletion can then be denoted 
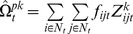
. If 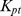 accounts for all feasible scenarios of *p*-arc deletion, the state of maximally inhibited nodal interaction is then induced by the scenario *k* where 
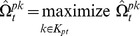
. Therefore, a lower bound on network robustness in epoch *t* is then the level of nodal interaction supported by the network (or non-inhibited) given a scenario of maximal disruption or 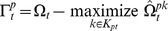 [22], [36]. In practice, the identification of arc deletion scenarios that provably 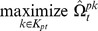 is a computational challenge, necessitating efficient optimization approaches and solution techniques [22], [30]–[31], [35]. In order to identify a bounding scenario of arc deletion, a mathematical programming problem can be structured based on the model of [22]. Let 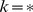 delineate the scenario of deletion resulting in maximal inhibition of nodal interaction to be sought and let 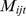 be the set of nodes *v* incident to node *i* through which node *j* can be reached via non-cyclic paths.
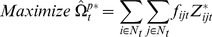
(1)Subject to:



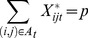
(2)

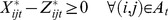
(3)

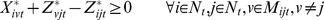
(4)

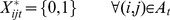
(5)

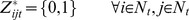
Model Objective (1) is to identify a network state where total nodal interaction is maximally inhibited. If the nodal interaction variable 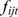 is omitted, Objective (1) would then be to maximally inhibit structural connectivity. Constraint (2) states that a scenario must be found that involves the deletion of exactly *p* arcs. Constraints (3) and (4) state that connectivity between a pair of nodes can only be completely inhibited if *no* paths between the nodal pair are available after arc deletion. That is, *any* path between a pair of nodes can provide connectivity until one or more of the arcs participating in the path are deleted. Given this constraint structure, *all* potential paths of movement between each nodal pair are considered in the modeling framework (not just the shortest or some subset of paths) [22]. Constraints (5) ensure that the decisions made by the model are binary-integer in nature. Given the linear-integer structure of this model, it can be solved using techniques such as branch-and-bound [37], available in many commercial optimization software packages. Once solved, the variables 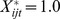 will indicate which arcs are selected for deletion and the variables 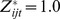 will indicate which nodal pairs are no longer connected given the optimal scenario of arc deletion. Network robustness to the most disruptive mechanism of *p*-arc deletion can then be reported as 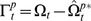.

Once a network’s robustness has been characterized, the sensitivity or elasticity of robustness to changes in the magnitude of arc deletion *p* can be evaluated. The elasticity of robustness in epoch *t* can be approximated as 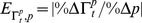 for any change in robustness relative to the corresponding change in *p* using standard midpoint elasticity calculations [38]. Given this formulation of elasticity, values greater than 1.0 represents increasing returns to scale, where changes in network robustness are very sensitive to changes in the magnitude of arc deletion. In other words, a larger elasticity indicates greater potential for a mechanism to efficiently degrade network performance. Conversely, robustness elasticity less than 1.0 indicates decreasing returns to scale, where changes in network robustness are less sensitive to changes in the magnitude of arc deletion. Simply put, a lower sensitivity means that the network can better withstand the effects of a disruptive mechanism.

## Results

**Figure 1:**
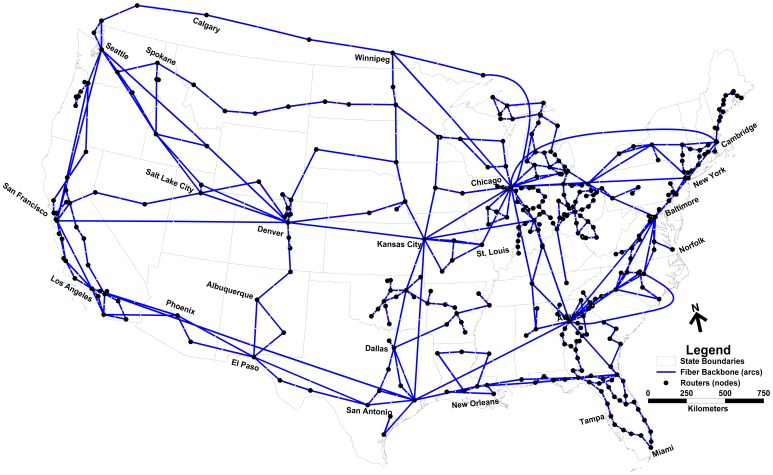
Internet2 backbone.

The concept of robustness elasticity is illustrated using the Internet2 backbone network for which observations of nodal interaction were recorded [39]. Topology and levels of nodal interaction for this Internet system were observed at network routers. When this study was conducted, 372 routers (nodes) and 495 fiber linkages (arcs) defined the backbone structure (Figure 1). Thus, in this network there are 138,384 nodal pairs (372×372) that can potentially interact with one another (sending/receiving data in this example). Data (i.e. bytes) transmitted between network nodes was recorded over a 24 hour period. Given the amount of data collected, network traffic is aggregated into six epochs for subsequent analysis. Table 1 shows a summary of network activity in the analysis epochs. Over the course of the day sampled, some level of data transmission is observed between 70,685 unique pairs of nodes (routers). Therefore, only around 51% of the nodal pairs in the network required connectivity on this day. The number of nodal pairs interacting varies considerably throughout the day with a high of 62,200 pairs engaged in the movement of nearly 24% of the day’s data in the 12 pm–4 pm epoch (all times are in U.S. Eastern Standard Time) to a low of 47,913 pairs supporting only 8% of the day’s interaction in the 4 am–8 am epoch (Table 1). In this paper, bounds on robustness for scenarios of arc deletion ranging from *p* = 1 (deletion of a single arc) to *p* = 20 (simultaneous deletion of 20 arcs) for each of the six epochs are sought. To accomplish this, a total of 120 instances to the optimization model (1)-(5) are generated to derive twenty bounding scenarios for each of the six epochs. Each of the 120 modeling instances is then solved to optimality using IBM’s ILOG CPLEX v12.1.0, a commercial optimization solver. Using the optimal deletion scenarios, the Internet network’s robustness to arc loss 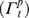 can then evaluated.

**Table 1:** Summary of observed nodal interaction by epoch.

Epoch	Interacting Node Pairs	% Daily Interaction
12 am–4 am	50,516	11
4 am–8 am	47,913	8
8 am–12 pm	60,168	19
12 pm–4 pm	62,202	24
4 pm–8 pm	59,741	22
8 pm–12 am	54,777	17
**Day Total**	70,685 Unique Pairs	100

**Figure 2:**
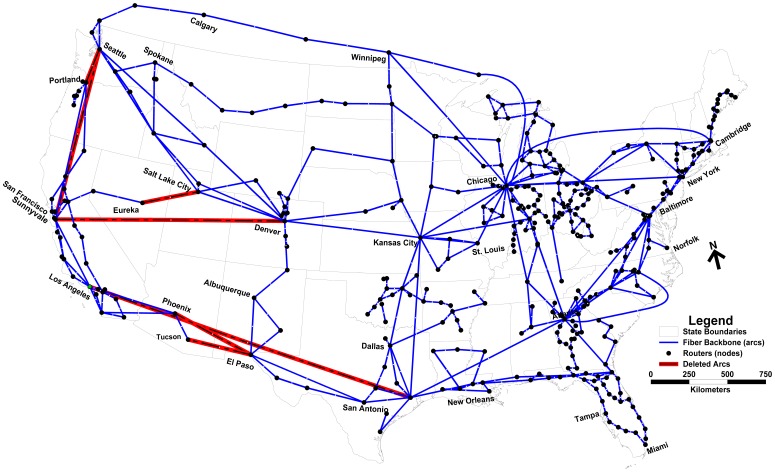
Seven arc deletion scenario bounding robustness 8 am–12 pm.

**Figure 3:**
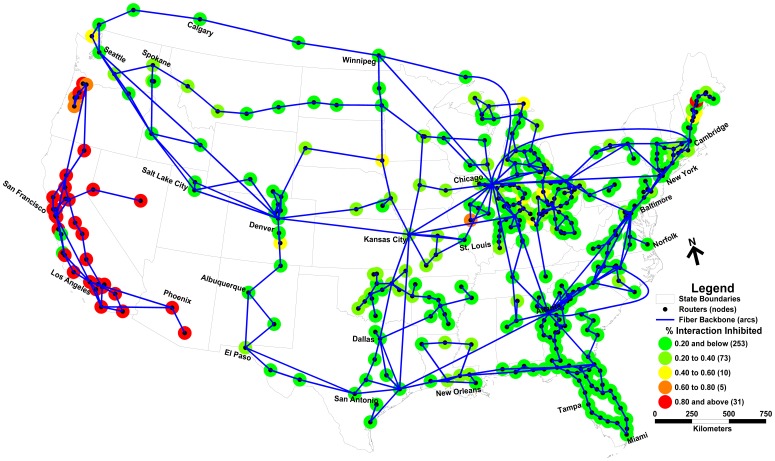
Maximally inhibited nodal interaction: 8 am–12 pm.

**Figure 4:**
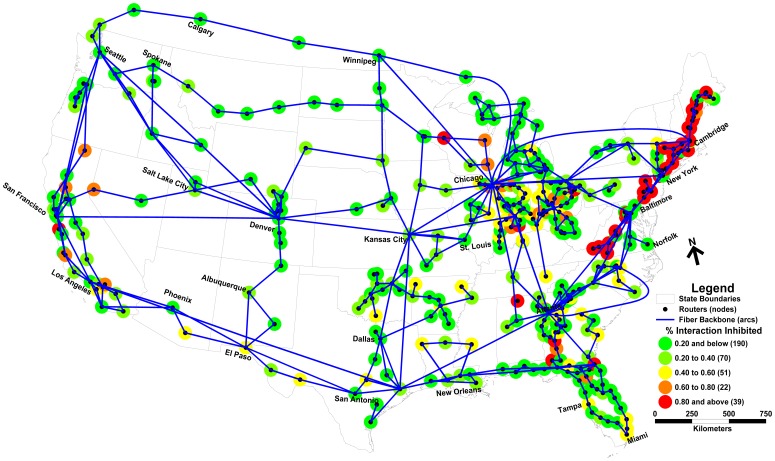
Maximally inhibited nodal interaction: 8 pm–12 am.

Table 2 details the bounds on robustness for each epoch for the different levels of *p*-arc deletion assessed. To better visualize the nature of these bounds, Figure 2 illustrates the scenario of seven arc deletion (*p = 7*) identified by the optimization approach as maximally inhibiting nodal interaction in the 8 am–12 pm epoch. The seven arcs comprising this optimal scenario constitute a cutset, fragmenting the network into two subgraphs. In this particular epoch, this cutset inhibits 33.7% of all nodal interaction (66.3% interaction is non-inhibited). Therefore, while nodes in the same subgraph (i.e. Denver and New York) are still connected and can interact, nodes in different subgraphs (i.e. New York and Los Angeles) are no longer connected and all interaction among them has been inhibited. In order to more clearly illustrate the spatial distribution of inhibited nodal interaction, Figures 3–4 depict the backbone network and the percent of interaction inhibited at each node (deleted arcs are not shown). Figure 3 details the level of nodal interaction inhibited in the 8 am–12 pm epoch due to the optimal deletion scenario of seven arcs shown in Figure 2. As highlighted in Figure 3, many nodes in California, Oregon, Nevada, and Arizona experience higher levels of disruption given that most of their interaction was with nodes in the Eastern subgraph. Many of the nodes in the Eastern subgraph display lower levels of disruption on average, indicating they required little or no connectivity with the Western subgraph in this epoch. Interestingly, there are some non-intuitive pockets of nodes elsewhere in the network (e.g. Maine) that too experience significantly degraded levels of interaction – likely a function of their demand for interaction with Western nodes during this epoch. Figure 4 depicts the percent degradation in nodal interaction at network nodes in the 8 pm–12 am epoch. In this case, the seven deleted arcs maximally inhibiting nodal interaction (36.9% of total interaction inhibited; 63.1% non-inhibited) are primarily located in the Northeastern portion of the network comprising a completely different scenario from that shown in Figure 2. The resulting spatial distribution of disruption varies considerably among network nodes. While interaction between many nodal pairs experiences little to no reduction, interaction among other nodes (i.e. those in the Northeastern region where arc deletion occurred) is severely diminished. Although very distant from the deleted arcs, significant levels of inhibited nodal interaction can be observed in Wisconsin, California, Georgia, as well as many other locales. While the *number* of arcs deleted in the 8 pm–12 am and 8 am–12 pm epochs is the same (*p* = 7), the *set* of arcs selected for deletion in each epoch are very different as is the impact of their deletion on nodal interaction. These changes in the role of the network arcs and in the spatial distribution of nodal interaction disrupted highlight the remarkable sensitivity of network robustness, both in time and space, to arc deletion. This finding is clear evidence that network robustness is indeed dependent on the spatial and temporal organization of interacting nodes within a network.

**Table 2:** Bounds on robustness (% non-inhibited nodal interaction) to *p*-arc deletion by epoch.

*p*	12 am–4 am	4 am–8 am	8 am–12 pm	12 pm–4 pm	4 pm–8 pm	8 pm–12 am
**0**	100.00	100.00	100.00	100.00	100.00	100.00
**1**	95.16	93.66	96.00	96.52	95.51	95.23
**2**	84.60	85.30	84.38	83.83	84.60	84.88
**3**	79.92	79.08	81.23	80.74	80.64	81.04
**4**	74.35	75.89	76.34	76.66	75.15	72.72
**5**	69.68	70.12	73.21	73.59	71.60	68.89
**6**	67.05	67.16	71.18	70.97	68.71	66.90
**7**	64.53	63.93	66.30	62.18	62.81	63.07
**8**	45.35	42.28	37.41	38.71	42.80	47.71
**9**	42.35	39.75	35.06	36.77	40.63	44.84
**10**	35.65	34.36	28.84	29.28	33.32	36.14
**11**	32.80	32.25	27.25	27.72	31.47	33.76
**12**	27.88	28.53	23.84	24.61	26.29	27.83
**13**	25.84	26.50	22.19	23.07	24.83	26.38
**14**	24.23	24.42	21.12	21.76	23.12	22.95
**15**	21.08	20.77	19.71	20.72	21.67	20.82
**16**	19.43	19.09	18.71	19.14	19.97	19.14
**17**	17.64	17.46	16.63	17.32	18.16	17.30
**18**	15.56	15.89	15.06	15.58	16.32	15.75
**19**	13.93	14.35	13.01	13.58	14.42	14.12
**20**	12.98	13.12	12.42	13.04	13.61	13.42

**Figure 5:**
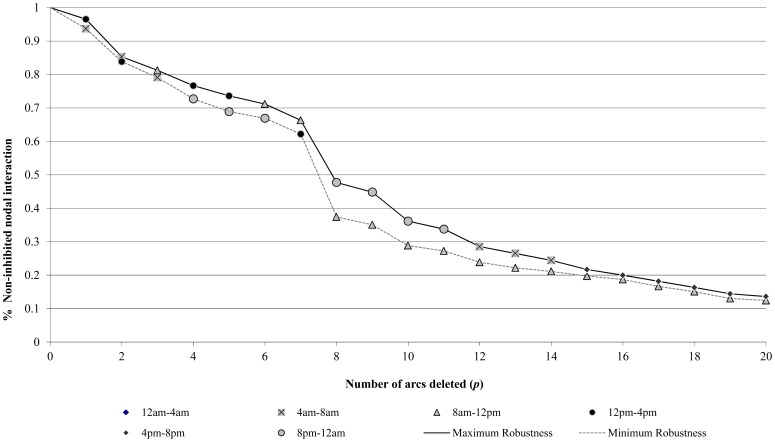
Epochs with maximum and minimum robustness for bounding scenarios of *p*-arc deletion.

Figure 5 summarizes the derived bounds on network robustness from Table 2 for those epochs most robust (maximum) and least robust (minimum) to optimal *p*-arc deletion. In this Figure, the robustness of the other four epochs, though not shown for clarity, falls somewhere between these extrema. Without identification of these bounds, the relationship of a deletion mechanism to a most disruptive or ‘worst-case’ mechanism is impossible to assess. As shown, the bounds on robustness can range significantly within each epoch. For example, given the simultaneous deletion of two arcs (*p* = 2), the 12 pm–4 pm epoch is when the network is least robust, respective to the other epochs, where 83.8% nodal interaction not inhibited. Conversely, the 4 am–8 am epoch is the most robust, where 85.3% interaction is not inhibited. Although the difference in robustness between these two epochs for *p* = 2 is rather small, it becomes more pronounced as the number of arcs deleted increases. Consider, for example, an eight arc deletion scenario (*p* = 8). In this instance, the network is most robust in the 8 pm–12 am epoch, where 47.7% of nodal interaction is not inhibited. However, the network is least robust to the deletion of eight arcs during the 8 am–12 pm epoch where only 37.4% nodal interaction is not inhibited. As expected, larger magnitudes of arc deletion (larger *p*) are met by lower levels of robustness within a single epoch. However, it is noted that this tendency does not necessarily carry over *between* epochs as depicted in multiple cases in Figure 5. For instance, network robustness is much higher for the deletion of nine arcs (*p* = 9) in the 8 pm–12 am epoch than it is for the loss of 8 arcs (*p* = 8) in the 8 am–12 pm epoch. This behavior is clear evidence of the influence that variations in nodal interaction can have on characterizations of robustness. In particular, changing patterns of nodal interaction can affect how the network is used to facilitate these interactions. Further, the arc deletion scenarios comprising the bounds on robustness can vary in arc composition both between *and* within epochs as highlighted in the previous example. That is to say that an arc contributing to a scenario of *p*-arc deletion bounding network robustness may not be included in bounding scenarios for other magnitudes of arc deletion given the interdependencies among network nodes.

**Figure 6:**
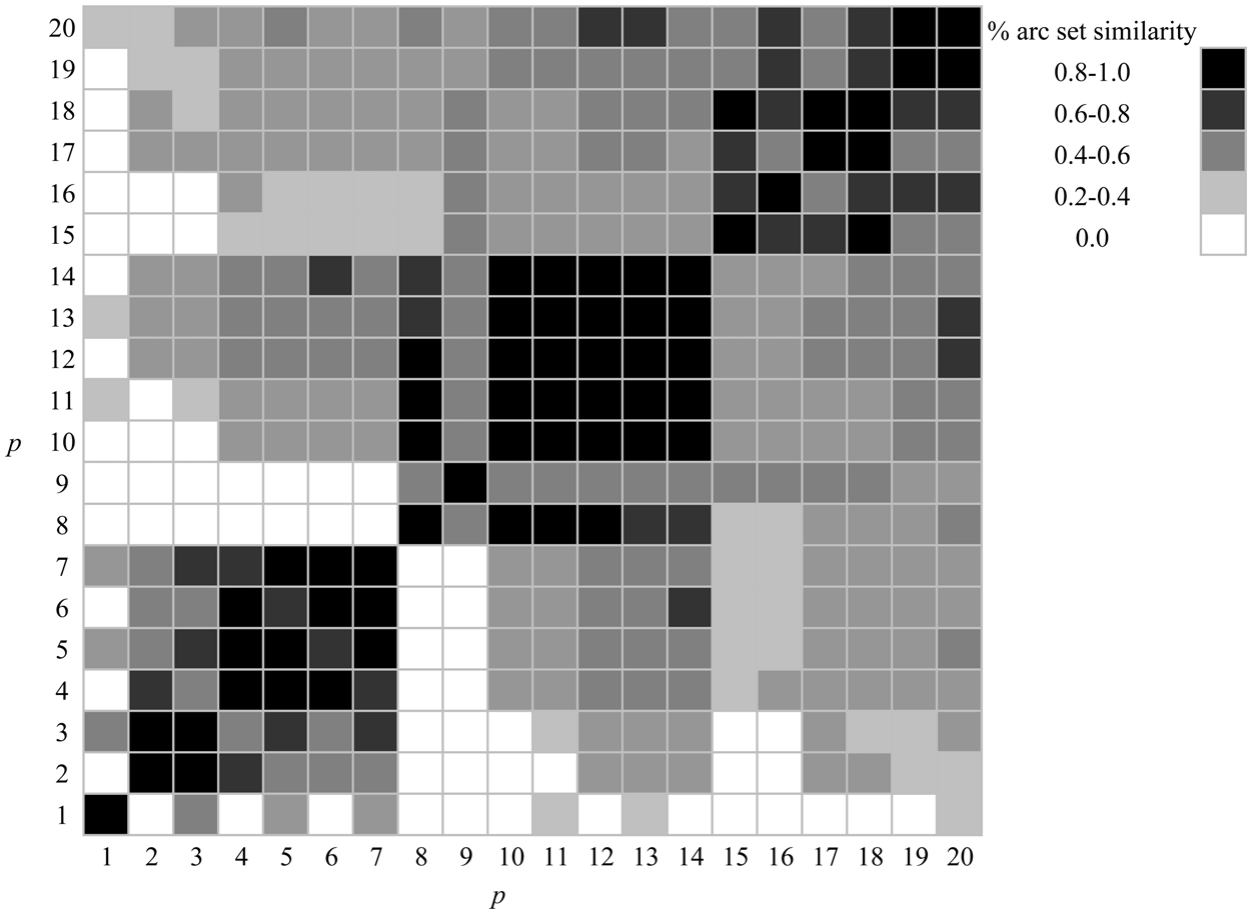
Similarity among sets of arcs comprising bounding deletion scenarios.

To better illustrate the relationship between the sets of arcs involved in bounding scenarios, Figure 6 displays Dice’s Coefficient [40], a measure of set similarity, for the sets of deleted arcs maximally inhibiting interaction in the 8 pm–12 am epoch. This matrix depicts the percent similarity in the arcs selected for deletion between any pair of bounding scenarios. For instance, row one of the matrix describes the extent to which the single arc deleted in the *p* = 1 scenario is also deleted in the other deletion scenarios. Thus, as detailed in row one of the matrix, the single arc deleted in the scenario bounding *p* = 1 is not selected for deletion in the scenarios bounding *p* = 2, 4, 6, 8–10, 12, and 14–19; hence, bearing no similarity with them. However, the arc selected for deletion in *p* = 1 is also selected for deletion in scenarios where *p* = 3, 5, 7, 11, 13, and 20. Row eight of the matrix indicates that none of the eight arcs deleted in the optimal *p* = 8 scenario are deleted in the *p* = 1–7 scenarios. Yet, many of the arcs selected for deletion in the *p* = 8 scenario are also selected for deletion in other bounding scenarios, such as is the case for *p* = 10–14 (at least 80% similarity in the arcs selected for deletion). Since the set of arcs characterizing robustness in one epoch may differ so significantly from those characterizing robustness in other epochs, these results provide further evidence that dynamics in nodal interaction give rise to variations in the importance of arcs to the network.

**Figure 7:**
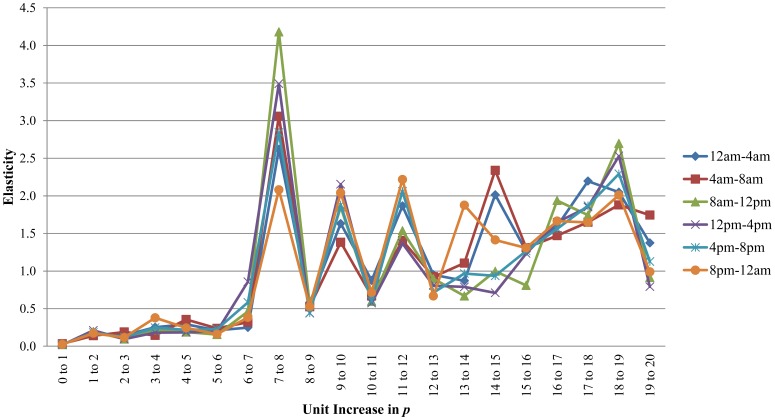
Robustness elasticity.

Figure 7 illustrates robustness elasticity for the network relative to each unit change in arc deletion magnitude over the six epochs of observed nodal interaction. In essence, this is a relative measure of robustness sensitivity to changes in magnitude of arc deletion (*p*). As illustrated by Figure 7, robustness elasticity (on the *y*-axis) varies substantially given unit increases in *p*. This particular network is relatively non-elastic (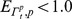) when arc deletion magnitudes are low (e.g. increase from *p* = 1 to *p* = 2 or from *p* = 6 to *p* = 7). One reason for this is that unit changes between smaller values of *p* will result in a greater percent change in disruptive magnitude when compared to unit changes between larger values of *p.* For example, when moving from *p* = 1 to *p* = 2, the network experiences a large percent decrease in robustness (over 11%). However, the percent change in *p* is also relatively large (over 66%), resulting in low elasticity. That said, most instances exhibit a much smaller change in robustness relative to the magnitude of arc deletion. The non-elastic nature of robustness at these magnitudes of arc deletion can be seen as indicative as greater network resistance to a disruptive mechanism or greater effort needed by the mechanism to maximally inhibit nodal interaction. However, an increase from *p* = 7 to *p* = 8 indicates a clear bifurcation point, where increasing returns to scale (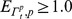) are realized. This greater elasticity can be viewed as a decrease in resistance to a particular mechanism of disruption or alternatively, as an increase in the efficiency with which a mechanism of disruption can degrade the network’s performance. In contrast to the previous example, this bifurcation is due to a relatively large percent change in robustness that accompanies a relatively small change in arc deletion magnitude between *p* = 7 to *p* = 8. In the 8 pm to 12 am epoch, robustness is decreased nearly 24% given an increase from *p* = 7 to *p* = 8. In the 8 am–12 pm epoch, robustness is decreased nearly 44% given an increase from *p* = 7 to *p* = 8. This is a major difference and again points to the dynamic nature of both networks and their robustness properties. While the network’s robustness in both periods is very sensitive to a small change in the number of arcs deleted, robustness in the 8 am–12 pm epoch displays a considerably higher level of sensitivity to this change. Although the 8 am–12 pm is the most robust to the deletion of seven arcs, this slight increase in the number of arcs deleted results in the 8 am–12 pm epoch being the least robust to a *p* = 8 scenario (Figure 5). In general, it is observed that after larger decrease in robustness, such as that accompanying the increase from *p* = 7 to *p* = 8, robustness elasticity tends to briefly diminish, fluctuating between non-elasticity and elasticity. As shown in Figure 7, there is considerable variation among the epochs as to which one is the most elastic or least elastic over the increases in *p* considered. In most cases though, all six epochs do together tend toward relative elasticity or non-elasticity for each increase in *p*. However, in a few instances, change in arc deletion magnitude results in elasticity in one epoch while resulting in relative non-elasticity in others.

## Discussion

To effectively capture and describe network robustness with respect to changes in the distribution of nodal interaction in a network, one significant challenge is to ensure that measures of robustness are consistent and comparable under the range of operational states experienced by a system. The identification of exact mathematical bounds on robustness facilitates unbiased comparisons of nodal interaction across different network states (i.e. epochs). However, the identification of these mathematical extrema (i.e. bounding scenarios) is difficult given the multifaceted and non-intuitive interdependencies defining complex networks. These exact bounds are essential for providing a comparative benchmark for other measures of network robustness identified through modeling other mechanisms of network change [18], [27]–[32]. This is particularly important since many mechanisms of network change have been proposed and in many cases, their characterizations of robustness relative to one another tend to lack consistency within and between networks [18], [27]–[28]. Thus, the ability to assess the proximity of a network’s robustness to any other mechanism of *p*-arc or node deletion relative to the bounding mechanism will certainly add great analytical strength in evaluations of robustness.

The modeling approach detailed in this paper complements existing work in several ways. First, it allows one to simultaneously evaluate network structure as well as performance (e.g. interaction inhibited), two of the most important facets of network robustness. Second, this approach considers all unique paths between nodal pairs when determining connectivity, eliminating the need to use simple approximations for connectivity (e.g. nodal degree) or to further generalize network structure. Computationally, the model structure also represents an improvement over similar structures given that it requires fewer constraints to represent the network, enhancing its ability to be solved to optimality. Since optimal solutions can be identified using this modeling framework, the results are consistent and provide an objective benchmark for all potential deletion scenarios. Finally, although applied to an Internet system in this paper, the modeling framework is system agnostic and can be adapted to any network.

Bounds on robustness to arc deletion are identified for a large Internet network for six different epochs of observed nodal interaction. While network structure is held constant over this sequence of network activity, the level of interaction among pairs of nodes varies considerably as does the robustness to arc deletion. The results presented here demonstrate that robustness can be highly elastic over the spatial and temporal dimensions of a network and is particularly sensitive to variations in nodal interaction. The results also indicate that the set of arcs selected for deletion in the bounding scenarios can be dramatically different in arc composition given different magnitudes of arc deletion as well as different states (i.e. epochs) of nodal interaction. This is a critical finding since it provides firm evidence that the ‘importance’ of an arc or node in a network cannot assumed to be constant over different magnitudes of deletion. This is particularly true in cases where nodal interaction is dynamic. Given that characterizations of robustness can display such spatial and temporal variation, it is imperative to carefully consider how nodal interaction within complex systems is represented in analyses and how the selected representation of interaction might impact the evaluation of network robustness. This is especially important when the analysis results are used to inform planning decisions on where to invest financial and human resources to improve a network’s robustness to arc and/or node deletion.
